# Experimental realization of controlled quantum teleportation of arbitrary qubit states via cluster states

**DOI:** 10.1038/s41598-020-70446-8

**Published:** 2020-08-12

**Authors:** Abhijeet Kumar, Saeed Haddadi, Mohammad Reza Pourkarimi, Bikash K. Behera, Prasanta K. Panigrahi

**Affiliations:** 1 Department of Physics, Central University of Jharkhand, Ranchi, Jharkhand 835205 India; 2 Faculty of Physics, Semnan University, P.O. Box 35195-363, Semnan, Iran; 3 Department of Physics, Salman Farsi University of Kazerun, Kazerun, Iran; 4 Bikash’s Quantum (OPC) Pvt. Ltd., Balindi, Mohanpur, Nadia, West Bengal 741246 India; 5 Department of Physical Sciences, Indian Institute of Science Education and Research Kolkata, Mohanpur, West Bengal 741246 India

**Keywords:** Physics, Quantum physics

## Abstract

Controlled quantum teleportation involves a third party as a controller for the teleportation of state. Here, we present the novel protocols for controlling teleportation of the arbitrary two-qubit and three-qubit states through five-qubit and seven-qubit cluster states respectively. In these schemes, Alice sends the arbitrary qubit states to the remote receiver Bob through the cluster states as quantum channels under the control of Charlie. Bob can recover the mentioned states by making appropriate unitary operations, and we point out that the efficiency in our schemes is 100%. In the process of our analysis, we find the classical communication cost in our protocols is remarkably reduced when compared to the previous protocols. We perform the experimental realization of the above protocols on “IBM 16 Melbourne” quantum computer and “IBM quantum simulator” and we calculate the fidelity. We also examine the security analysis against Charlie, and these schemes which we considered here are secure against Charlie’s attacks.

## Introduction

Following the idea of Bennett et al.^1,2^ on quantum teleportation, we use entanglement^3^ for the quantum communication protocols^4–19^. Such as teleportation of qubits^20^, quantum key distribution (QKD)^21,22^, quantum secret sharing^23,24^, etc.^25–34^ Entanglement can be seen in many states likes Bell states^35,36^, GHZ states^37^, and W states^38,39^, and so far several measures have been proposed to quantify entanglement^40–45^. Various research works have been developed in the field of multi-party quantum teleportation^46–48^. As far as we know, the first quantum teleportation between three parties is proposed by Karlsson et al.^49^ in 1998 using GHZ state. Also, Dong et al.^50^ performed a controlled communication between the three-party using GHZ state and imperfect Bell state measurement. Furthermore, Hassanpour et al.^51^ performed controlled quantum secure direct communication protocol using GHZ-like states.

Quantum teleportation involving cluster states^7,52–55^ is a multiparty protocol. Cluster states are a kind of highly entangled quantum states, and they can be prepared in the following ways: (a) Cluster states can be generated in lattices of spin qubits by interacting them with “Ising type Hamiltonian”^56^, (b) Cluster states can be generated by spontaneous parametric down-conversion involving photon polarization and non-linear optics^57^. (c) The cluster states are considered as a particular case of graph states^58–61^. Cluster states have great importance over quantum teleportation, and they can be used for one-way quantum computing^55,57^, bidirectional quantum computing^62^, and cyclic quantum computing^63^. In our protocols, we use the cluster states as a one-way quantum computing channel.

Quantum correlation is used as a resource to establish entanglement between the particles. For an entangled state, the entanglement of formation^42^ specifies the amount of resource used to generate the particular entanglement between the particles. The amount of resource used for generating entanglement between particles is referred to as quantum entanglement cost^64^. Besides, the quality of a quantum circuit is measured by the number of gates used in the circuit. So the quantum cost of a circuit is defined as the number of preliminary gates used in the circuit. In fact, as the number of gates decreases, the cost of the circuit will be reduced.

More recently, Haddadi et al.^65^ proposed a protocol for teleportation of the two-qubit state through a five-qubit cluster state. Remarkably, they have shown that their protocol is deterministic, i.e. the probability of success in their scheme is 100%. We propose the protocols involving five-qubit and seven-qubit cluster states for teleportation of two-qubit and three-qubit states respectively. In our protocols, we use cluster states as quantum channels shared between three parties Alice, Bob and Charlie. Where Alice (sender) sends her qubits information to Bob (receiver) under control, supervision of Charlie (controller) through the shared quantum channel between them.

Till date, no one has examined our protocols for teleportation of the arbitrary two-qubit and three-qubit states through the five-qubit and seven-qubit cluster states as quantum channels. Thus we are motivated to analyze them theoretically as well as experimentally. Herein, we use IBM Quantum Experience (IBM QE)^8,66–80^ platform for the experimental realization of the quantum circuits. Indeed, IBM QE is an online service that allows access to the most advanced quantum computers for the researcher to do research work and run quantum programs on IBM Q systems with the IBM QE cloud platform. There are some processors on IBM QE such as one 1-qubit processor, six 5-qubit processors, one 15-qubit processor, and one 32-qubit simulator. In this work, we use “IBM 16 Melbourne” quantum computer and “IBM qasm simulator” for experimental realization of our quantum circuits.

This paper comprises of various sections as follows. The next section explains the theoretical and experimental approach of the controlled quantum teleportation protocols for five-qubit and seven-qubit cluster states. In “Quantum state tomography” section is devoted to quantum state tomography and the “Results” section discusses the results of the proposed protocols by showing the fidelity of the circuits. In “Security analysis against Charlie” section discusses the security analysis against Charlie’s attacks. Finally, we end our paper with a brief conclusion in the last section.

## Theoretical and experimental realization of our protocols

There are many schemes for the controlled quantum teleportation^81,82^. In the following sub-sections, we discuss the controlled quantum teleportation of arbitrary qubit states via cluster states for two cases. Case I: scheme for the controlled quantum teleportation of an arbitrary two-qubit state using a five-qubit cluster state. Case II: scheme for the controlled quantum teleportation of an arbitrary three-qubit state using a seven-qubit cluster state.

**Figure 1:**
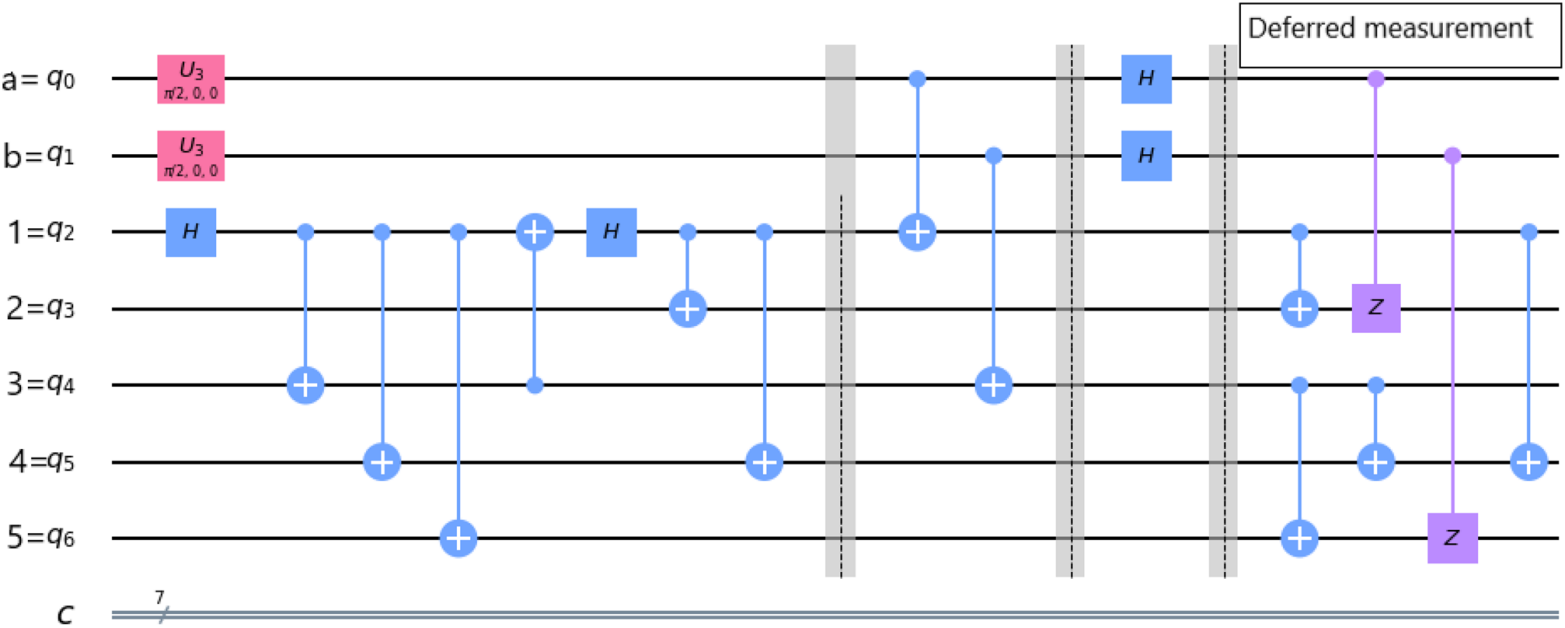
Alice performs the necessary unitary operations on arbitrary two-qubit and five-qubit cluster state.

**Figure 2:**
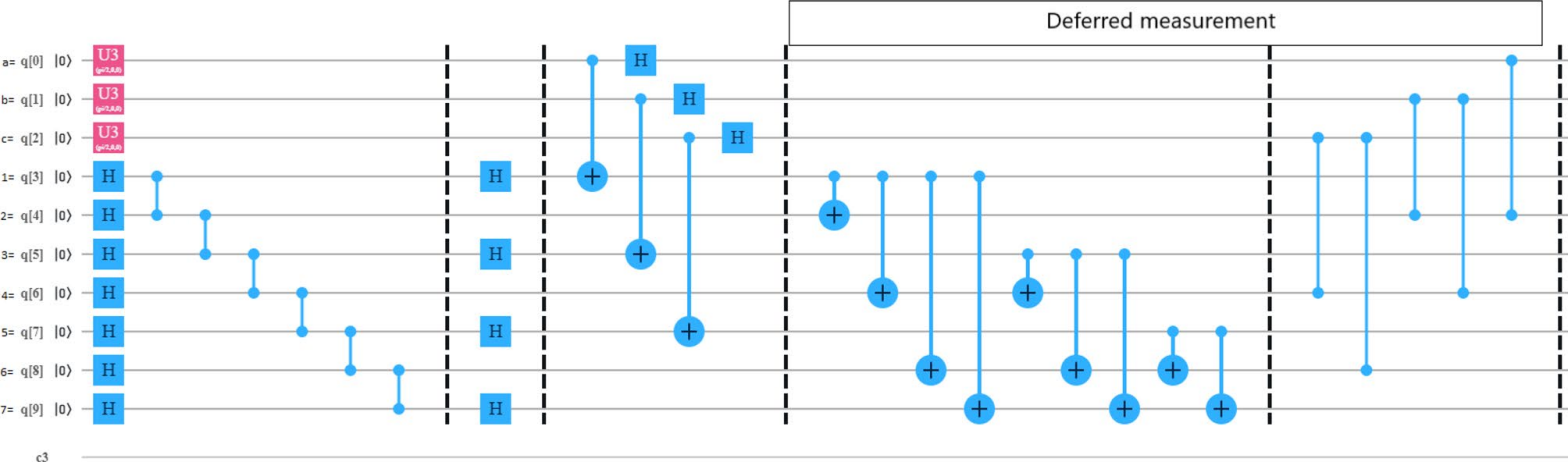
Alice performs the necessary unitary operations on arbitrary three-qubit and seven-qubit cluster state.

In our protocols, the cluster state has been remotely prepared at the Alice place, where she performs all the necessary unitary operations including the deferred measurement^83^ as shown in Figs. 1 and 2. After performing all the operations, Alice sends the respective qubits to the respective parties. Then Alice immediate measures her qubits in computational basis, which destroys her qubits, making her incapable of any further communication^55^. Now, after receiving qubits from Alice, Charlie measures his qubits in $| \pm \rangle$ basis ($| \pm \rangle =(| 0 \rangle \pm | 1 \rangle )/\sqrt{2}$). If the measurement outcome is $| - \rangle$, then Charlie sends a classical bit of information to Bob within a certain time period. After receiving Charlie’s classical information in a certain time period, Bob gets that Charlie’s measurement outcome is $| - \rangle$, and he has to perform a set of unitary operations on his qubits. If the measurement outcome is $| + \rangle$, then Charlie does not need to send any classical information to Bob. After waiting for a certain time period and have not received any classical information, Bob understands that Charlie’s measurement outcome is $| + \rangle$ and he has to perform another set of unitary operations on his qubits. The unitary operations, which Bob performs are discussed in the later sub-sections. By the above analysis, we conclude that the classical communication is taking place between Charlie and Bob, only when Charlie’s measurement outcome is $| - \rangle$. Hence, the average classical communication cost^84^ necessary for our protocols is 0.5 bit. In Table 1, we compared the classical communication cost of our protocols with the results of the other protocols.

**Table 1:** The comparison of classical cost between our protocols and others protocols. Quantum state: the remotely prepared state which is send to receiver’s end. Quantum channel state: The state used to teleport the quantum state. Classical cost: The minimum classical communication cost required to send the quantum state.

No.	Protocol	Quantum state	Quantum channel state	Classical cost
1	Shi et al.^85^	$\alpha | 00 \rangle + \beta | 11 \rangle$	GHZ state	1 Bit
2	Liu et al.^86^	$\alpha | 00 \rangle + \beta | 11 \rangle$	Bell state	2 Bits
3	Dai et al.^87^	$\alpha | 0000 \rangle + \beta | 1111 \rangle$	GHZ state	1 Bit
4	Zhan^88^	$\alpha | 00 \rangle + \beta | 11 \rangle$	Bell state	2 Bits
5	Liu et al.^89^	$\alpha | 000 \rangle + \beta | 111 \rangle$	Bell and GHZ states	0.5 Bit
6	Pan et al.^84^	$\alpha \prod _{i=1}^{m}| 0 \rangle _{i}+\beta \prod _{i=1}^{m}| 1 \rangle _{i}$	Bell state	0.5 Bit
7	Our protocol	$| \psi \rangle _{ab}$	5-qubit cluster state (1)	0.5 Bit
8	Our protocol	$| \chi \rangle _{abc}$	7-qubit cluster state (10)	0.5 Bit

### Scheme for controlled quantum teleportation of an arbitrary two-qubit state using a five-qubit cluster state

We consider a five-qubit cluster state which we used as a quantum channel to teleport a two-qubit state. The cluster state is shared between three parties Alice, Bob and Charlie which are far apart from each other. Alice shares two qubits, Charlie shares one qubit, and Bob shares the remaining two qubits of the five-qubit entangled cluster state. The average classical information shared between Charlie and Bob is 0.5 bit, which depends upon the measurement outcome of Charlie. Now, Bob takes the information of the classical channel into account and decides which set of unitary operations he has to perform on his qubits. Here, if Charlie wants to cheat and sends the wrong information to Bob through the classical channel, then after Bob’s unitary operation and consulting with Alice, Bob finds out that Charlie cheated. Thus, even if Charlie wants to, he cannot cheat without getting caught, and this is the beauty of quantum communication^23^.

**Figure 3:**
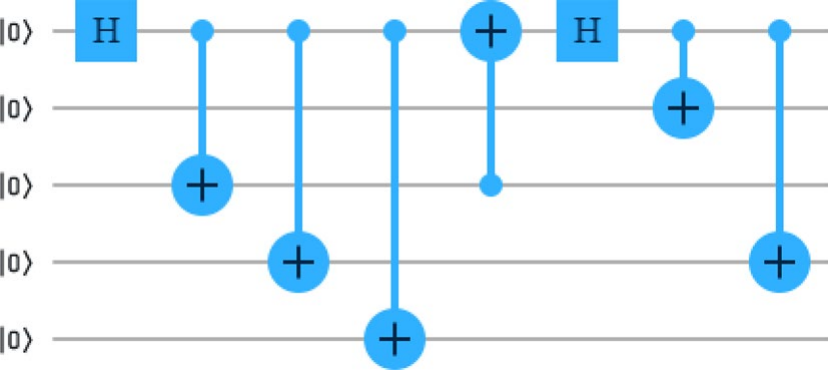
Quantum circuit generating the five-qubit cluster state, $|C_5\rangle _{12345}$.

The five-qubit cluster state from state $| 00000 \rangle _{12345}$ is generated by the following circuit as shown in Fig. 3, is used here as a quantum channel for the quantum communication between Alice, Bob and Charlie. Given as

1 $$\begin{aligned} | C_5 \rangle _{12345}={\frac{1}{2}(| 00000 \rangle + | 00111 \rangle + | 11010 \rangle + | 11101 \rangle )_{12345}}. \end{aligned}$$

In this scheme, we wish to teleport any two-qubit state $| \psi \rangle _{ab} =(\alpha | 00 \rangle +\beta | 01 \rangle +\gamma | 10 \rangle +\delta | 11 \rangle )_{ab}$ through the five-qubit cluster state (1), where $|\alpha |^2+|\beta |^2+|\gamma |^2+|\delta |^2=1$. The qubits 1 and 3 belong to Alice, the qubit 4 belongs to Charlie, and the qubits 2 and 5 belong to Bob. Now, the joint state of the arbitrary two-qubit state and the five-qubit cluster state can be written as $| \psi \rangle _{ab12345} =| \psi \rangle _{ab} \otimes | C_5 \rangle _{12345}$.

**Figure 4:**
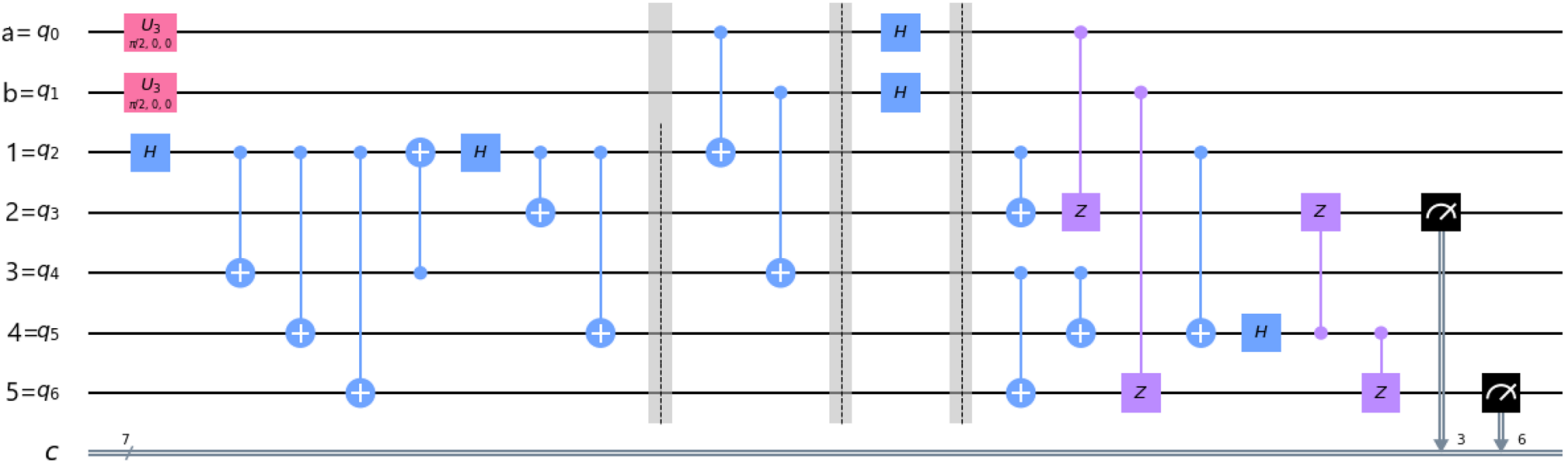
A generalized circuit for teleporting arbitrary two-qubit state using five-qubit cluster state.

Briefly, in this process of teleportation. Alice remotely prepared the cluster state at her place, and then she performs controlled-NOT (CNOT) gate on qubits (*a*, 1) and qubits (*b*, 3). Where the qubits *a* and *b* work as controlling qubits and qubits 1 and 3 work as target qubits (we use the abbreviation (*x*, *y*) where the qubit *x* works as controlling qubit and the qubit *y* works as target qubit, henceforth). After then, Alice performs Hadamard gate on her qubits *a* and *b*, and then she performs the deferred measurement on her qubits as discussed earlier. Then Alice sends the respective qubits to Charlie and Bob. If the controller Charlie agrees to help the communication between Alice and Bob, Charlie has to perform a single-qubit measurement in $| \pm \rangle$ basis on his qubit 4. Finally, Bob can obtain the unknown state by performing the appropriate set of unitary transformations on his qubits 2 and 5. Indeed, Fig. 4 shows the generalized equivalent circuit for teleporting two-qubit state by using a five-qubit cluster state.

#### Circuit decomposition

The arbitrary two-qubit state which Alice wishes to teleport is given as $| \psi \rangle _{ab} = (\alpha | 00 \rangle +\beta | 01 \rangle +\gamma | 10 \rangle +\delta | 11 \rangle )_{ab}$. The five-qubit cluster state which Alice used as a quantum channel for teleportation of two-qubit state is given as $| C_5 \rangle _{12345}$ in Eq. (1). Now, Alice implements arbitrary two-qubit to her share of entangled qubits in following ways: First, Alice performs CNOT gate on qubits (*a*, 1) and qubits (*b*, 3), and the joint state $| \psi \rangle _{ab12345}$ is changed to

2 $$\begin{aligned} | \psi ' \rangle _{ab12345}&= 1/2[\alpha | 00 \rangle _{ab}\otimes | \phi ^1 \rangle +\beta | 01 \rangle _{ab}\otimes | \phi ^2 \rangle \\&\quad +\gamma | 10 \rangle _{ab}\otimes | \phi ^3 \rangle + \delta | 11 \rangle _{ab}\otimes | \phi ^4 \rangle ], \end{aligned}$$

where

3 $$\begin{aligned}&| \phi ^1 \rangle = (| 00000 \rangle +| 00111 \rangle +| 11010 \rangle +| 11101 \rangle )_{12345},\\&| \phi ^2 \rangle = (| 00100 \rangle +| 00011 \rangle +| 11110 \rangle +| 11001 \rangle )_{12345},\\&| \phi ^3 \rangle = (| 10000 \rangle +| 10111 \rangle +| 01010 \rangle +| 01101 \rangle )_{12345},\\&| \phi ^4 \rangle = (| 10100 \rangle +| 10011 \rangle +| 01110 \rangle +| 01001 \rangle )_{12345}.\end{aligned}$$

Next, Alice applies Hadamard gate on her qubits *a* and *b* and the new state is given as

4 $$\begin{aligned} | \psi '' \rangle _{ab12345}&= 1/4[\alpha (| 00 \rangle +| 01 \rangle +| 10 \rangle +| 11 \rangle )_{ab} \otimes | \phi ^1 \rangle \\&\quad +\beta (| 00 \rangle -| 01 \rangle +| 10 \rangle -| 11 \rangle )_{ab}\otimes | \phi ^2 \rangle \\&\quad +\gamma (| 00 \rangle +| 01 \rangle -| 10 \rangle -| 11 \rangle )_{ab}\otimes | \phi ^3 \rangle \\&\quad +\delta (| 00 \rangle -| 01 \rangle -| 10 \rangle +| 11 \rangle )_{ab}\otimes | \phi ^4 \rangle ]. \end{aligned}$$

After expanding and rearranging the above equation, we have

5 $$\begin{aligned} | \psi '' \rangle _{ab12345}& = {} 1/4[| 0000 \rangle _{ab13}\otimes | \psi ^1 \rangle + | 0001 \rangle _{ab13}\otimes | \psi ^2 \rangle +| 0010 \rangle _{ab13}\otimes | \psi ^3 \rangle \\&\quad +| 0011 \rangle _{ab13}\otimes | \psi ^4 \rangle +| 0100 \rangle _{ab13}\otimes | \psi ^5 \rangle + | 0101 \rangle _{ab13}\otimes | \psi ^6 \rangle \\&\quad +| 0110 \rangle _{ab13}\otimes | \psi ^7 \rangle +| 0111 \rangle _{ab13}\otimes | \psi ^8 \rangle +| 1000 \rangle _{ab13}\otimes | \psi ^9 \rangle \\&\quad +| 1001 \rangle _{ab13}\otimes | \psi ^{10} \rangle +| 1010 \rangle _{ab13}\otimes | \psi ^{11} \rangle +| 1011 \rangle _{ab13}\otimes | \psi ^{12} \rangle \\&\quad + | 1100 \rangle _{ab13}\otimes | \psi ^{13} \rangle +| 1101 \rangle _{ab13}\otimes | \psi ^{14} \rangle +| 1110 \rangle _{ab13}\otimes | \psi ^{15} \rangle \\&\quad +| 1111 \rangle _{ab13}\otimes | \psi ^{16} \rangle ], \end{aligned}$$

where

6 $$\begin{aligned} | \psi ^1 \rangle& = {} (\alpha | 000 \rangle +\beta | 011 \rangle +\gamma | 101 \rangle +\delta | 110 \rangle )_{254}, \\ | \psi ^2 \rangle& = {} (\alpha | 011 \rangle +\beta | 000 \rangle +\gamma | 110 \rangle +\delta | 101 \rangle )_{254}, \\ | \psi ^3 \rangle& = {} (\alpha | 101 \rangle +\beta | 110 \rangle +\gamma | 000 \rangle +\delta | 011 \rangle )_{254}, \\ | \psi ^4 \rangle& = {} (\alpha | 110 \rangle +\beta | 101 \rangle +\gamma | 011 \rangle +\delta | 000 \rangle )_{254}, \\ | \psi ^5 \rangle& = {} (\alpha | 000 \rangle -\beta | 011 \rangle +\gamma | 101 \rangle -\delta | 110 \rangle )_{254}, \\ | \psi ^6 \rangle& = {} (\alpha | 011 \rangle -\beta | 000 \rangle +\gamma | 110 \rangle -\delta | 101 \rangle )_{254}, \\ | \psi ^7 \rangle& = {} (\alpha | 101 \rangle -\beta | 110 \rangle +\gamma | 000 \rangle -\delta | 011 \rangle )_{254}, \\ | \psi ^8 \rangle& = {} (\alpha | 110 \rangle -\beta | 101 \rangle +\gamma | 011 \rangle -\delta | 000 \rangle )_{254}, \\ | \psi ^9 \rangle& = {} (\alpha | 000 \rangle +\beta | 011 \rangle -\gamma | 101 \rangle -\delta | 110 \rangle )_{254}, \\ | \psi ^{10} \rangle& = {} (\alpha | 011 \rangle +\beta | 000 \rangle -\gamma | 110 \rangle -\delta | 101 \rangle )_{254}, \\ | \psi ^{11} \rangle& = {} (\alpha | 101 \rangle +\beta | 110 \rangle -\gamma | 000 \rangle -\delta | 011 \rangle )_{254}, \\ | \psi ^{12} \rangle& = {} (\alpha | 110 \rangle +\beta | 101 \rangle -\gamma | 011 \rangle -\delta | 000 \rangle )_{254}, \\ | \psi ^{13} \rangle& = {} (\alpha | 000 \rangle -\beta | 011 \rangle -\gamma | 101 \rangle +\delta | 110 \rangle )_{254}, \\ | \psi ^{14} \rangle& = {} (\alpha | 011 \rangle -\beta | 000 \rangle -\gamma | 110 \rangle +\delta | 101 \rangle )_{254}, \\ | \psi ^{15} \rangle& = {} (\alpha | 101 \rangle -\beta | 110 \rangle -\gamma | 000 \rangle +\delta | 011 \rangle )_{254}, \\ | \psi ^{16} \rangle& = {} (\alpha | 110 \rangle -\beta | 101 \rangle -\gamma | 011 \rangle +\delta | 000 \rangle )_{254}. \end{aligned}$$

To make the teleportation successful and also to reduce the classical communication cost, we use the deferred measurement^83^. After using the deferred measurement, the average classical communication cost required for quantum teleportation is 0.5 bit. In the deferred measurement, CNOT gate is applied on qubits (1, 2), (3, 5), (3, 4), (1, 4) and controlled-Z (CZ) gate is applied on qubits (*a*, 2) and (*b*, 5). After applying the deferred measurements, the state (5) becomes,

7 $$\begin{aligned} | \psi ''' \rangle _{ab12345}&= \frac{1}{4}(| 0000 \rangle +| 0001 \rangle +| 0010 \rangle +| 0011 \rangle +| 0100 \rangle +| 0101 \rangle +| 0110 \rangle \\&\quad +| 0111 \rangle +| 1000 \rangle +| 1001 \rangle +| 1010 \rangle +| 1011 \rangle +| 1100 \rangle +| 1101 \rangle \\&\quad +| 1110 \rangle +| 1111 \rangle )_{ab13}\otimes (\alpha | 000 \rangle +\beta | 011 \rangle +\gamma | 101 \rangle +\delta | 110 \rangle )_{254}. \end{aligned}$$

Alice measured her sets of qubits in the computational basis, then the whole state is collapsed to $| \phi \rangle _{254} = (\alpha | 000 \rangle +\beta | 011 \rangle +\gamma | 101 \rangle +\delta | 110 \rangle )_{254}$ and this can be written in the form as

8 $$\begin{aligned} | \phi \rangle _{254}& = {} (\alpha | 000 \rangle +\beta | 011 \rangle +\gamma | 101 \rangle +\delta | 110 \rangle )_{254} \\& = {} \frac{1}{\sqrt{2}}(\alpha | 00 \rangle +\beta | 01 \rangle +\gamma | 10 \rangle +\delta | 11 \rangle )_{25}\otimes | + \rangle _{4} \\&\quad + \frac{1}{\sqrt{2}}(\alpha | 00 \rangle -\beta | 01 \rangle -\gamma | 10 \rangle +\delta | 11 \rangle )_{25}\otimes | - \rangle _{4}, \end{aligned}$$

Charlie measures his qubit in $|+\rangle _{4}$ or $|-\rangle _{4}$ basis, and when his measured state is $| - \rangle _4$, then Charlie sends his qubit information in one bit to Bob within a certain time period through a secure classical channel. After receiving the information from Charlie, Bob gets that Charlie’s qubit is in $| - \rangle_4$ state, and he has to apply a phase-change unitary transformation on his qubits. If Charlie measurement outcome is $| + \rangle _{4}$, then he does not have to send any classical information to Bob. Whereas Bob after waiting for a certain time period and have not received any information from Charlie, he understands that Charlie’s qubit is in $| + \rangle_4$ state. And he has to apply an identity gate unitary transformation on his qubits (see Table 2). For example, let us say Alice measures her state and it comes out $| 0111 \rangle$, then the whole state is get collapsed to Eq. (8). After receiving qubits from Alice, Charlie measures his qubit state and let us say that the measurement outcome is $| - \rangle_4$. Then Charlie sends a 1 bit of classical information to Bob. Then Bob understands that he has to perform a phase-change unitary transformation on his qubits, means he applies *Z* gate on both of his qubits 2 and 5 to get the state sent by Alice. So this protocol is deterministic, i.e. the probability of success is 100%.

**Table 2:** Classical communication and unitary operations.

C.M.S	C. I	B.U.O 2nd qubit	B.U.O 5th qubit
$| + \rangle$	No classical information has been sent	I	I
$| - \rangle$	1 Bit	Z	Z

*C.M.S.* Charlie’s measured state of his qubit, *C.I:* classical information sent from Charlie to Bob, *B.U.O.* Bob applying unitary operations on his qubits.

We perform the above experiment on IBM QE, where we compare the statistical data of the teleportation of two-qubit between “IBM qasm-simulator” (with 32 qubits) and the “IBM 16 Melbourne” (IBM 16 Melbourne is a real device with 15 qubits). Indeed, we send an arbitrary two-qubit state from Alice to Bob with the help of a controller Charlie. We also figure out the density matrix of both the cases and from density matrices, we evaluate the fidelity^90,91^ of the circuit.

### Scheme for controlled quantum teleportation of an arbitrary three-qubit state using a seven-qubit cluster state

**Figure 5:**
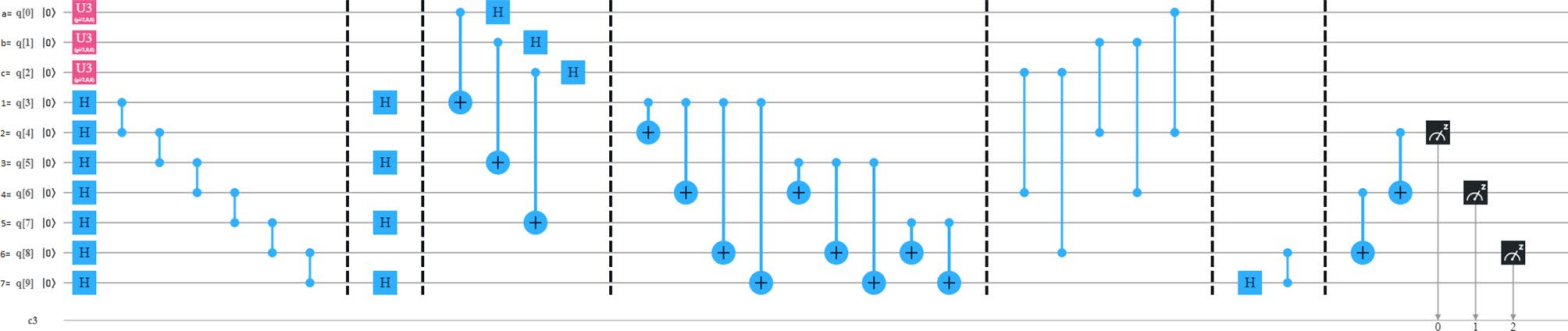
A generalized circuit for teleporting arbitrary three-qubit state using seven-qubit cluster state, $|C_7\rangle _{1234567}$.

In this case, we consider a seven-qubit cluster state which we used as a quantum channel for teleportation of three-qubit state. The cluster state is shared between as usual Alice (sender), Bob (receiver), and Charlie (controller) which are far apart from each other. More precisely, Alice and Bob each share three qubits and Charlie shares one qubit of the seven-qubit cluster state. The procedure is the same as for the previous scheme, i.e. Alice remotely prepared the cluster state at her place. After applying the required unitary operations between the cluster state and the three-qubit state, Alice sends the respective qubits to the respective parties. And after that, she immediately measured her qubits state and the whole state is get collapsed to Eq. (16). Next, Charlie measures his qubit in $|+\rangle _{7}$ or $|-\rangle _{7}$ basis, and when his measured state is $| - \rangle _7$, then Charlie sends his qubit information in one bit to Bob within a certain time period through a secure classical channel. After receiving the information from Charlie, Bob gets that Charlie’s qubit is in $| - \rangle_7$ state, and he has to apply a phase-change unitary transformation on qubit 6 followed by CNOT operation on qubits (2, 4) and (4, 6). If Charlie measurement outcome is $| + \rangle _7$, then he does not have to send any classical information to Bob. Whereas Bob after waiting for a certain time period and have not received any information from Charlie, he gets that Charlie’s qubit is in $| + \rangle_7$ state. And he has to apply an identity gate unitary transformation on qubit 6 followed by CNOT operation on qubits (2, 4) and (4, 6). Herein, the qubits 1, 3 and 5 belong to Alice, the qubits 2, 4 and 6 belong to Bob, and the qubit 7 belongs to Charlie. Indeed, Fig. 5 shows the generalized equivalent circuit for teleporting three-qubit state by using a seven-qubit cluster state.

#### Circuit decomposition

The arbitrary three-qubit state which Alice wish to teleport is given as

9 $$\begin{aligned} | \chi \rangle _{abc}&= (\alpha | 000 \rangle +\beta | 001 \rangle +\gamma | 010 \rangle +\delta | 011 \rangle \\&\quad +\epsilon | 100 \rangle +\zeta | 101 \rangle +\eta | 110 \rangle +\theta | 111 \rangle )_{abc}, \end{aligned}$$

where $$|\alpha |^2 + |\beta |^2 + |\gamma |^2 + |\delta |^2 + |\epsilon |^2 + |\zeta |^2 + |\eta |^2 + |\theta |^2 = 1$$.

**Figure 6:**
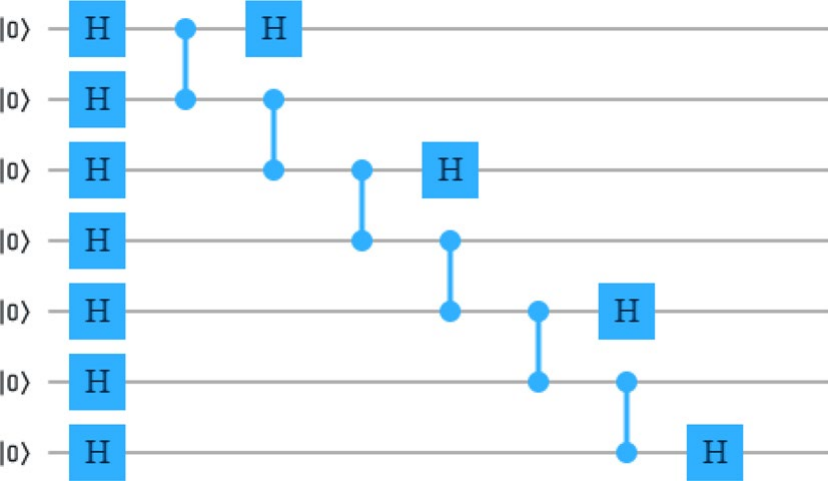
Quantum circuit generating the seven-qubit cluster state, $|C_7\rangle _{1234567}$.

The seven-qubit cluster state from state $| 0000000 \rangle _{1234567}$ is generated by the following circuit as shown in Fig. 6, is used as a quantum channel for teleportation of the three-qubit state reads

10 $$\begin{aligned} | C_7 \rangle _{1234567}&= \frac{1}{2\sqrt{2}}(| 0000000 \rangle + | 1110000 \rangle +| 1101100 \rangle +| 0011100 \rangle \\&\quad +| 1101011 \rangle +| 0011011 \rangle +| 0000111 \rangle +| 1110111 \rangle )_{1234567}. \end{aligned}$$

The joint state of the arbitrary three-qubit state and the seven-qubit cluster state can be written as $| \chi \rangle _{abc1234567} =| \chi \rangle _{abc}\otimes | C_7 \rangle _{1234567}$, i.e.

11 $$\begin{aligned} | \chi \rangle _{abc1234567}&= [(\alpha | 000 \rangle +\beta | 001 \rangle +\gamma | 010 \rangle +\delta | 011 \rangle +\epsilon | 100 \rangle +\zeta | 101 \rangle \\&\quad +\eta | 110 \rangle +\theta | 111 \rangle )\otimes \frac{1}{2\sqrt{2}}(| 0000000 \rangle + | 1110000 \rangle +| 1101100 \rangle \\&\quad +| 0011100 \rangle +| 1101011 \rangle +| 0011011 \rangle +| 0000111 \rangle +| 1110111 \rangle )]_{abc1234567}. \end{aligned}$$

Now, Alice implements arbitrary three-qubit to her share of entangled qubits in following ways: **Step 1** Considering the initial joint state $| \chi \rangle _{abc1234567}$, Alice applies CNOT gate on her qubits (*a*, 1), (*b*, 3), and (*c*, 5). Therefore, the transformed state is given as

12 $$\begin{aligned} | \chi ' \rangle _{abc1234567}&= \frac{1}{2\sqrt{2}}[\alpha | 000 \rangle \otimes (| 0000000 \rangle + | 1110000 \rangle +| 1101100 \rangle +| 0011100 \rangle \\&\quad +| 1101011 \rangle +| 0011011 \rangle +| 0000111 \rangle +| 1110111 \rangle )\\&\quad +\beta | 001 \rangle \otimes (| 0000100 \rangle +| 1110100 \rangle + | 1101000 \rangle +| 0011000 \rangle \\&\quad +| 1101111 \rangle +| 0011111 \rangle +| 0000011 \rangle +| 1110011 \rangle )\\&\quad +\gamma | 010 \rangle \otimes (| 0010000 \rangle +| 1100000 \rangle +| 1111100 \rangle +| 0001100 \rangle \\&\quad +| 1111011 \rangle +| 0001011 \rangle +| 0010111 \rangle +| 1100111 \rangle )\\&\quad +\delta | 011 \rangle \otimes (| 0010100 \rangle +| 1100100 \rangle + | 1111000 \rangle +| 0001000 \rangle \\&\quad +| 1111111 \rangle +| 0001111 \rangle +| 0010011 \rangle +| 1100011 \rangle )\\&\quad +\epsilon | 100 \rangle \otimes (| 1000000 \rangle +| 0110000 \rangle +| 0101100 \rangle +| 1011100 \rangle \\&\quad +| 0101011 \rangle +| 1011011 \rangle +| 1000111 \rangle +| 0110111 \rangle )\\&\quad +\zeta | 101 \rangle \otimes (| 1000100 \rangle +| 0110100 \rangle + | 0101000 \rangle +| 1011000 \rangle \\&\quad +| 0101111 \rangle +| 1011111 \rangle +| 1000011 \rangle +| 0110011 \rangle )\\&\quad +\eta | 110 \rangle \otimes (| 1010000 \rangle +| 0100000 \rangle +| 0111100 \rangle +| 1001100 \rangle \\&\quad +| 0111011 \rangle +| 1001011 \rangle +| 1010111 \rangle +| 0100111 \rangle )\\&\quad +\theta | 111 \rangle \otimes (| 1010100 \rangle +| 0100100 \rangle +| 0111000 \rangle +| 1001000 \rangle \\&\quad +| 0111111 \rangle +| 1001111 \rangle +| 1010011 \rangle +| 0100011 \rangle )]_{abc1234567}. \end{aligned}$$

**Step 2** Alice applies Hadamard gate on her qubits *a*, *b* and *c* and after that, the deferred measurement is performed on the circuit in a systematic way. Now in the deferred measurement, CNOT gate and CZ gate applied on qubits, i.e. Alice performs CNOT gate on qubits (1, 2), (1, 4), (1, 6), (1, 7), (3, 4), (3, 6), (3, 7), (5, 6), (5, 7), and then she performs CZ gate on qubits (*c*, 4), (*c*, 6), (*b*, 2), (*b*, 4), (*a*, 2). After all the operations, the new state is obtained as

13 $$\begin{aligned} | \chi '' \rangle _{abc1234567}&= \frac{1}{8}\sum _{l=0}^{l=63}| \chi _l \rangle _{abc135} \otimes (\alpha | 0000 \rangle +\beta | 0011 \rangle +\gamma | 0111 \rangle +\delta | 0100 \rangle \\&\quad +\epsilon | 1111 \rangle +\zeta | 1100 \rangle +\eta | 1000 \rangle +\theta | 1011 \rangle )_{2467}, \end{aligned}$$

where

14 $$\begin{aligned} \frac{1}{8}\sum _{l=0}^{l=63}{| \chi _l \rangle }_{abc135} = \frac{1}{8}(| \chi _0 \rangle +| \chi _1 \rangle +| \chi _2 \rangle +| \chi _3 \rangle +\cdots +| \chi _{62} \rangle +| \chi _{63} \rangle )_{abc135}, \end{aligned}$$

and

15 $$\begin{aligned} | \chi _0 \rangle&=| 000000 \rangle ,\;\;| \chi _1 \rangle =| 000001 \rangle ,\;\;| \chi _2 \rangle =| 000010 \rangle ,\;\;| \chi _3 \rangle =| 000011 \rangle ,\;\;\\ | \chi _4 \rangle&=| 000100 \rangle ,\;\;| \chi _5 \rangle =| 000101 \rangle ,\;\;| \chi _6 \rangle =| 000110 \rangle ,\;\;| \chi _7 \rangle =| 000111 \rangle ,\;\;\\ | \chi _8 \rangle&=| 001000 \rangle ,\;\;| \chi _9 \rangle =| 001001 \rangle ,\;\; | \chi _{10} \rangle =| 001010 \rangle ,\;| \chi _{11} \rangle =| 001011 \rangle ,\;\\ | \chi _{12} \rangle&=| 001100 \rangle ,\;| \chi _{13} \rangle =| 001101 \rangle ,\; | \chi _{14} \rangle =| 001110 \rangle ,\;| \chi _{15} \rangle =| 001111 \rangle ,\;\\ | \chi _{16} \rangle&=| 010000 \rangle ,\;| \chi _{17} \rangle =| 010001 \rangle ,\; | \chi _{18} \rangle =| 010010 \rangle ,\;| \chi _{19} \rangle =| 010011 \rangle ,\;\\ | \chi _{20} \rangle&=| 010100 \rangle ,\;| \chi _{21} \rangle =| 010101 \rangle ,\; | \chi _{22} \rangle =| 010110 \rangle ,\;| \chi _{23} \rangle =| 010111 \rangle ,\;\\ | \chi _{24} \rangle&=| 011000 \rangle ,\;| \chi _{25} \rangle =| 011001 \rangle ,\; | \chi _{26} \rangle =| 011010 \rangle ,\;| \chi _{27} \rangle =| 011011 \rangle ,\;\\ | \chi _{28} \rangle&=| 011100 \rangle ,\;| \chi _{29} \rangle =| 011101 \rangle ,\; | \chi _{30} \rangle =| 011110 \rangle ,\;| \chi _{31} \rangle =| 011111 \rangle ,\;\\ | \chi _{32} \rangle&=| 100000 \rangle ,\;| \chi _{33} \rangle =| 100001 \rangle ,\; | \chi _{34} \rangle =| 100010 \rangle ,\;| \chi _{35} \rangle =| 100011 \rangle ,\;\\ | \chi _{36} \rangle&=| 100100 \rangle ,\;| \chi _{37} \rangle =| 100101 \rangle ,\; | \chi _{38} \rangle =| 100110 \rangle ,\;| \chi _{39} \rangle =| 100111 \rangle ,\;\\ | \chi _{40} \rangle&=| 101000 \rangle ,\;| \chi _{41} \rangle =| 101001 \rangle ,\; | \chi _{42} \rangle =| 101010 \rangle ,\;| \chi _{43} \rangle =| 101011 \rangle ,\;\\ | \chi _{44} \rangle&=| 101100 \rangle ,\;| \chi _{45} \rangle =| 101101 \rangle ,\; | \chi _{46} \rangle =| 101110 \rangle ,\;| \chi _{47} \rangle =| 101111 \rangle ,\;\\ | \chi _{48} \rangle&=| 110000 \rangle ,\;| \chi _{49} \rangle =| 110001 \rangle ,\; | \chi _{50} \rangle =| 110010 \rangle ,\;| \chi _{51} \rangle =| 110011 \rangle ,\;\\ | \chi _{52} \rangle&=| 110100 \rangle ,\;| \chi _{53} \rangle =| 110101 \rangle ,\; | \chi _{54} \rangle =| 110110 \rangle ,\;| \chi _{55} \rangle =| 110111 \rangle ,\;\\ | \chi _{56} \rangle&=| 111000 \rangle ,\;| \chi _{57} \rangle =| 111001 \rangle ,\; | \chi _{58} \rangle =| 111010 \rangle ,\;| \chi _{59} \rangle =| 111011 \rangle ,\;\\ | \chi _{60} \rangle&=| 111100 \rangle ,\;| \chi _{61} \rangle =| 111101 \rangle ,\; | \chi _{62} \rangle =| 111110 \rangle ,\;| \chi _{63} \rangle =| 111111 \rangle .\; \end{aligned}$$

**Step 3** In the following, Alice measured her states in a computational basis and Charlie measured his state in $| \pm \rangle$ basis. Then Charlie decides whether to send his qubit information to Bob or not depends upon his qubit state, as discussed earlier. Bob after getting the information decides which set of unitary operations, he has to apply on his qubits.

Now, if Alice measures her states then, as usual, the whole state (13) is going to collapse to $| \phi \rangle _{2467}=(\alpha | 0000 \rangle +\beta | 0011 \rangle +\gamma | 0111 \rangle +\delta | 0100 \rangle +\epsilon | 1111 \rangle +\zeta | 1100 \rangle +\eta | 1000 \rangle +\theta | 1011 \rangle )_{2467}$ and this can be written in the following form

16 $$\begin{aligned} | \phi \rangle _{2467}&= \frac{1}{\sqrt{2}}(\alpha | 000 \rangle +\beta | 001 \rangle +\gamma | 011 \rangle +\delta | 010 \rangle +\epsilon | 111 \rangle \\&\quad +\zeta | 110 \rangle +\eta | 100 \rangle +\theta | 101 \rangle )_{246} \otimes | + \rangle _{7}\\&\quad + \frac{1}{\sqrt{2}}(\alpha | 000 \rangle -\beta | 001 \rangle -\gamma | 011 \rangle +\delta | 010 \rangle -\epsilon | 111 \rangle \\&\quad +\zeta | 110 \rangle +\eta | 100 \rangle -\theta | 101 \rangle )_{246}\otimes | - \rangle _{7}. \end{aligned}$$

**Step 4** Now if Charlie’s measurement outcome is $| + \rangle _{7}$, then Bob has to perform first an identity gate on qubit 6 and then CNOT gate on qubits (4, 6) and (2, 4) to get the state $| \chi \rangle _{abc}$ as Alice wants to send. But if Charlie’s measurement outcome is $| - \rangle _{7}$, then Bob has to perform first a phase-change unitary transformation on qubit 6 and then CNOT gate on qubits (4, 6) and (2, 4) to get the initial state $| \chi \rangle _{abc}$ (see Table 3).

For example, let us say Alice measures her state and it comes out $| \chi _9 \rangle$. Now Charlie measured his state and if his measurement outcome is $| - \rangle _{7}$, then Bob understands that he has to perform a set of unitary transformation on his qubits to get the state, as discussed earlier. As a result, our protocol is deterministic, i.e. the probability of success achieves 100%.

**Table 3:** Classical communication and unitary operations.

C.M.S	C. I	B.U.O $2^{nd}$ Qubit	B.U.O $4^{th}$ Qubit	B.U.O $6^{th}$ Qubit
$| + \rangle$	No classical information has been sent	CNOT (2, 4)	CNOT (4, 6)	I
$| - \rangle$	1 Bit	CNOT (2, 4)	CNOT (4, 6)	Z

*C.M.S.* Charlie’s measured state of his qubit, *C.I.* classical information sent from Charlie to Bob, *B.U.O.* Bob applying unitary operations on his qubits.

We perform the above experiment in IBM QE, where we compare the statistical data of the teleportation of three-qubit state between “IBM qasm-simulator” and “IBM 16 Melbourne”. In this experiment, we send an arbitrary three-qubit state from Alice to Bob with the help of a controller Charlie. We run the experiment on both the simulator and the real device and from there we figure out the density matrix of both the cases and from density matrices, we evaluate the fidelity^90,91^ of the circuit.

## Quantum state tomography

Quantum state tomography is an approach to specify a quantum state which embraces the collation of theoretical and experimental density matrices. The theoretical density matrix of the quantum state prepared in the first instance is given by

17 $$\begin{aligned} \rho ^T = | \psi \rangle \langle \psi |. \end{aligned}$$

**Figure 7:**
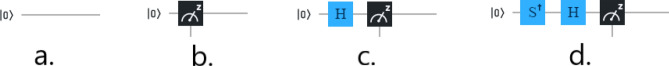
Measurement in different bases: (**a**) *I* basis, (**b**) *Z* basis, (**c**) *X* basis, (**d**) *Y* basis.

On the other hand, the experimental density matrix for *N*-qubit system is given as^67,69,92,93^

18 $$\begin{aligned} \rho ^E = \frac{1}{2^N}\sum _{j_1,j_2,j_3\ldots j_N=0}^{3}T_{j_1j_2j_3\ldots j_N}(\sigma _{j_1}\otimes \sigma _{j_2}\otimes \sigma _{j_3}\otimes \ldots \otimes \sigma _{j_N}), \end{aligned}$$

where $\sigma _{j_i}$ with $i \in \{1, 2,\ldots ,N\}$ is the Pauli matrix acting on *i*-th qubit. And $T_{j_1j_2j_3 \ldots j_N}$ represents the result of a particular measurement as

19 $$\begin{aligned} T_{j_1j_2j_3 \ldots j_N} = S_{j_1}\times S_{j_2}\times S_{j_3}\times \ldots \times S_{j_N}. \end{aligned}$$

where $S_{j_1}, S_{j_2}, S_{j_3}\ldots S_{j_N}$ are the Stokes parameters^67^, and $j_1, j_2 \ldots j_N$ are the indices and can take values 0, 1, 2 and 3 which represent the quantum gates *I*, *X*, *Y* and *Z* respectively. The Stokes parameters are $S_0 = P_{| 0I \rangle } + P_{| 1I \rangle }$, $S_1 = P_{| 0X \rangle } - P_{| 1X \rangle }$, $S_2 = P_{| 0Y \rangle } - P_{| 1Y \rangle }$, $S_3 = P_{| 0Z \rangle } - P_{| 1Z \rangle }$. Here, $P_{| 0I \rangle }$ represents the probability of the qubit to be found in $| 0 \rangle$ state when it is measured in *I* basis, and $P_{| 1I \rangle }$ represents the probability of the qubit to be found in $| 1 \rangle$ state when it is measured in *I* basis. The other notations $(P_{| 0X \rangle }, P_{| 1X \rangle })$, $(P_{| 0Y \rangle }, P_{| 1Y \rangle })$, and $(P_{| 0Z \rangle }, P_{| 1Z \rangle })$ held the same meaning, but instead of *I* basis, they are measured in *X*, *Y* and *Z* bases respectively (see Fig. 7).

## Results

In this section, we present the experimental results for both the cases. Namely the teleportation of an arbitrary two-qubit state using a five-qubit cluster state and then teleportation of an arbitrary three-qubit state using a seven-qubit cluster state.

### For teleportation of two-qubit state

**Figure 8:**
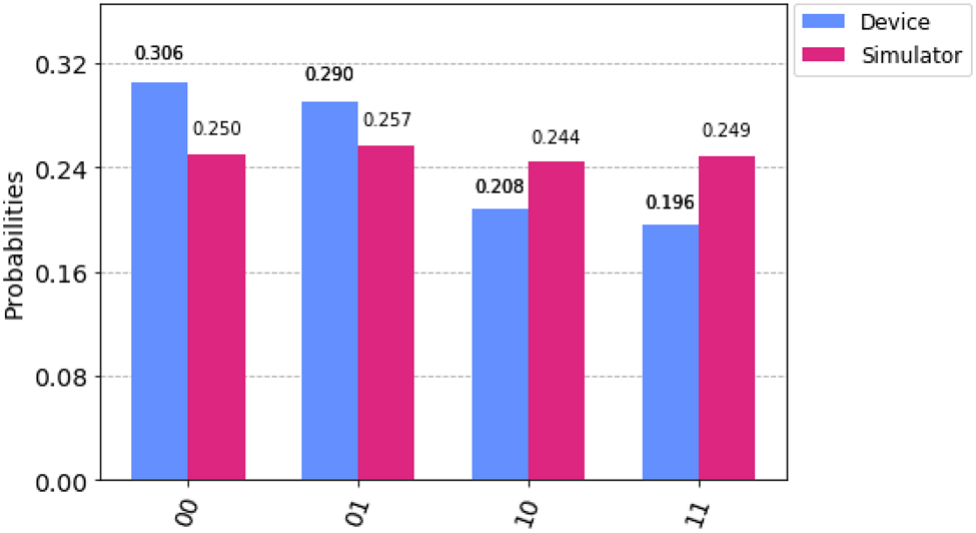
Histogram. Comparison between the probabilities obtained by “IBM qasm simulator” and “IBM 16 Melbourne” for teleportation of two-qubit state.

First, we run our circuit on “IBM qasm simulator” (with 8192 shots for more accuracy and to reduce statistical errors) and on “IBM 16 Melbourne” (real device). Then we compare both results as seen in Fig. 8. We observe that there are some errors in the “IBM 16 Melbourne” results, and these are due to decoherence^66^, state preparation, and also due to the number of gates used in the circuit^92^ as each gate inherently contains gate errors.

Now, we check the fidelity of the circuit so that we can know how well the state is teleported. Fidelity is given by the following formula^90,91^

20 $$\begin{aligned} F(\rho ^T, \rho ^E) =Tr \sqrt{\sqrt{\rho ^T}\rho ^E\sqrt{\rho ^T}}, \end{aligned}$$

here $\rho ^T$ is the theoretical density matrix whereas, $\rho ^E$ is the experimental density matrix, and for a positive semidefinite matrix *M*, $\sqrt{M}$ denotes its unique positive square root. The general equation of density matrix is given by Eq. (17). Now let us analyze a two-qubit state which is given by

21 $$\begin{aligned} | \psi \rangle _{ab} = \frac{1}{{2}}(| 00 \rangle +| 01 \rangle +| 10 \rangle +| 11 \rangle )_{ab}, \end{aligned}$$

for this case $\rho ^T$ is obtained as

22 $$\begin{aligned} \rho ^T = \frac{1}{4} \begin{pmatrix} 1 &{} 1 &{} 1 &{} 1\\ 1 &{} 1 &{} 1 &{} 1\\ 1 &{} 1 &{} 1 &{} 1\\ 1 &{} 1 &{} 1 &{} 1\\ \end{pmatrix} \end{aligned}$$

**Figure 9:**
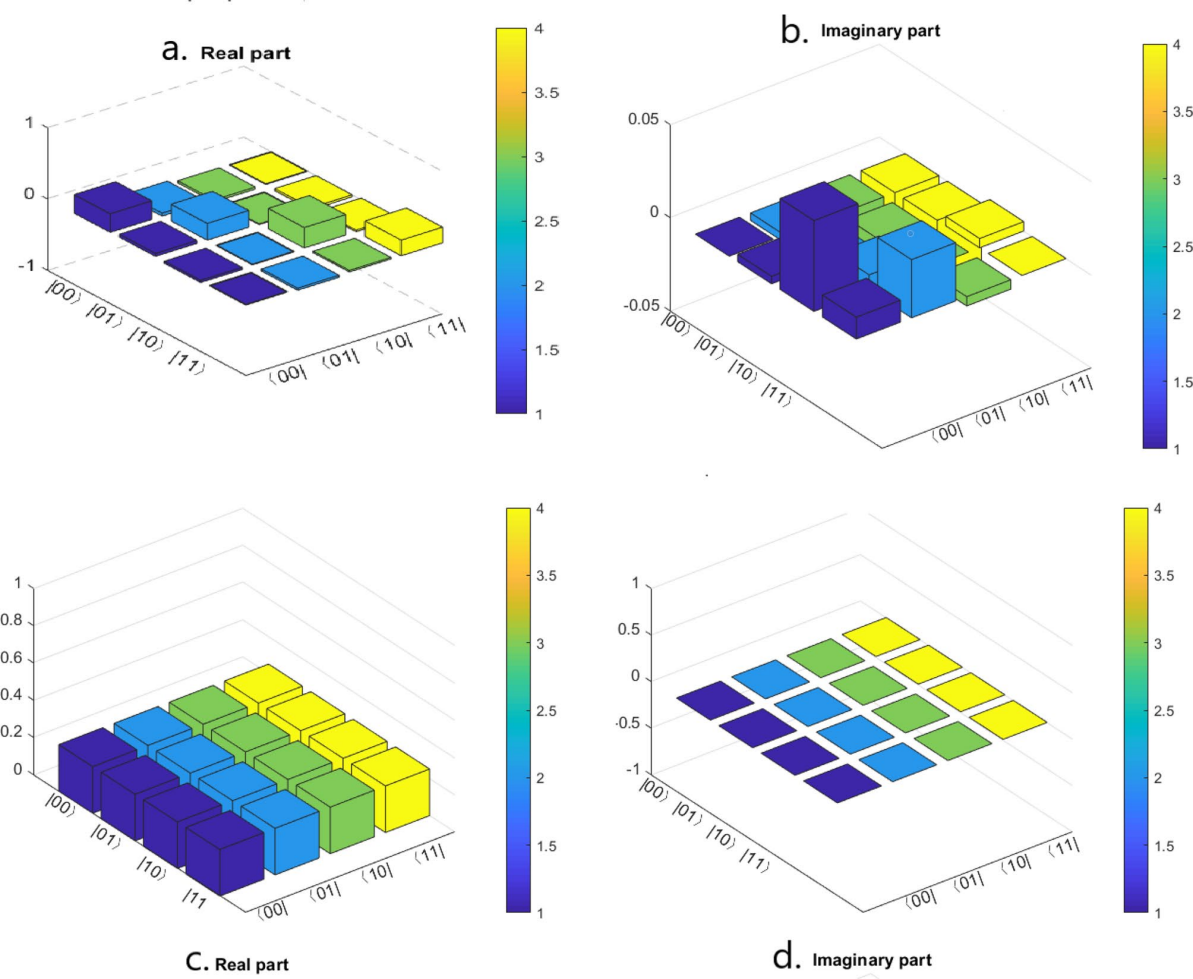
Real and imaginary parts of experimental and theoretical density matrices for teleportation of two-qubit state $| \psi \rangle _{ab}=\frac{1}{2}(| 00 \rangle +| 01 \rangle +| 10 \rangle +| 11 \rangle )_{ab}$. (**a**) Real part of the experimental density matrix, (**b**) imaginary part of the experimental density matrix, (**c**) real part of the theoretical density matrix, (**d**) imaginary part of the theoretical density matrix. These results are taken from the “IBM 16 Melbourne” device.

Besides, the experimental density matrix (18) for two qubits is given by the following formula^67,93^

23 $$\begin{aligned} \rho ^E = \frac{1}{4}\sum _{j_1,j_2=0}^{3}T_{j_1 j_2}(\sigma _{j_1}\otimes \sigma _{j_2}), \end{aligned}$$

here $T_{j_1j_2}$ is defined as $T_{j_1j_2} =S_{j_1}\times S_{j_2}$ where the Stokes parameters are $S_0 = P_{| 0I \rangle } + P_{| 1I \rangle }$, $S_1 = P_{| 0X \rangle } - P_{| 1X \rangle }$, $S_2 = P_{| 0Y \rangle } - P_{| 1Y \rangle }$, $S_3 = P_{| 0Z \rangle } - P_{| 1Z \rangle }$. Here, $P_{| 0j_1 \rangle }$ represents the probability of the qubit to be found in $| 0 \rangle$ state when it is measured in $j_1$ basis. $P_{| 1j_1 \rangle }$ represents the probability of the qubit to be found in $| 1 \rangle$ state when it is measured in $j_1$ basis, and same thing with $j_2$ basis. So, for a two-qubit state (21), the experimental density matrix is calculated as what follows (see Fig. 9)

24 $$\begin{aligned} \rho ^E = \begin{pmatrix} 0.2603 &{} 0.0410 &{} -0.0300 &{} -0.0100\\ 0.0410 &{} 0.2347 &{} -0.0100 &{} -0.0290\\ -0.0300 &{} -0.0100 &{} 0.2858 &{} 0.0220\\ -0.0100 &{} -0.0290 &{} 0.0220 &{} 0.2192 \end{pmatrix} +i \begin{pmatrix} 0 &{} -0.0040 &{} -0.0487 &{} -0.0118\\ 0.0040 &{} 0 &{} -0.0082 &{} -0.0312\\ 0.0487 &{} 0.0082 &{} 0 &{} 0.0050\\ 0.0118 &{} 0.0312 &{} -0.0050 &{} 0 \end{pmatrix} \end{aligned}$$

Finally, the fidelity between the theoretical density matrix (22) and the experimental density matrix (24) is calculated to be $F(\rho ^T, \rho ^E) = 0.4919$.

### For teleportation of three-qubit state

**Figure 10:**
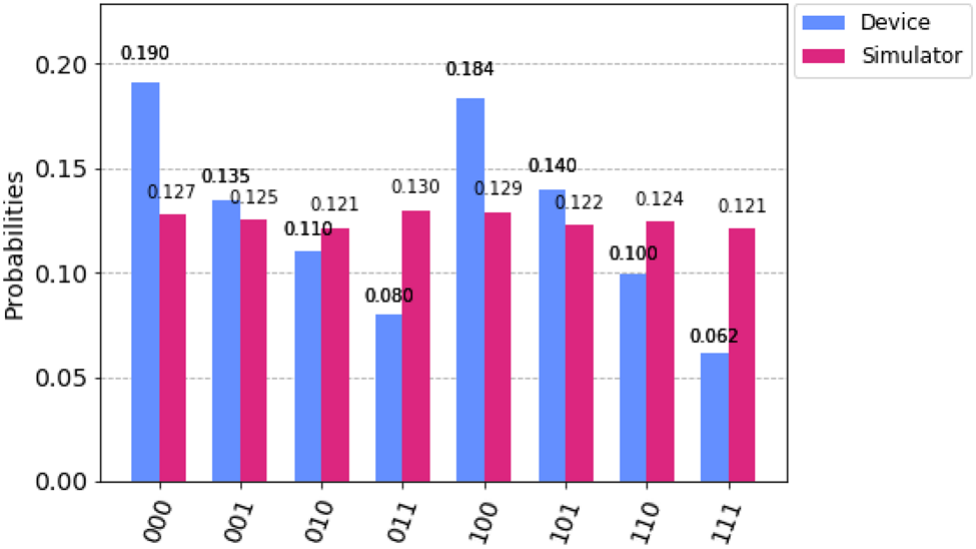
Histogram. Comparison between the probabilities obtained by “IBM qasm simulator” and “IBM 16 Melbourne” for teleportation of three-qubit state.

Here we perform the same as we did for two-qubit teleportation, i.e. we run our experiment on “IBM qasm simulator” (with 8192 shots for more accuracy) and on “IBM 16 Melbourne” (real device). As we can see in Fig. 10, that the probability of getting each possible states for the three-qubit system is nearly the same in the case of “IBM qasm simulator”. However, in the case of “IBM 16 Melbourne” we can observe that the probability of getting each possible states for the three-qubit system is different and this is due to the noise errors present in the quantum channel. These noise errors are due to decoherence^66^ in the quantum channel, state preparation error, and gate errors. Indeed, all these factors play an important role in reducing the fidelity of the states.

Now let us consider a three-qubit state as follows

25 $$\begin{aligned} | \chi \rangle _{abc} = \frac{1}{2\sqrt{2}}(| 000 \rangle +| 001 \rangle +| 010 \rangle +| 011 \rangle +| 100 \rangle +| 101 \rangle +| 110 \rangle +| 111 \rangle )_{abc}. \end{aligned}$$

In the previous section, we presented the general equation of the density matrix in Eq. (17) and then we defined fidelity in Eq. (20). Thus, for this state (25) the theoretical density matrix is obtained as

26 $$\begin{aligned} \rho ^T=\frac{1}{8}\begin{pmatrix} 1&{}1&{}1&{}1&{}1&{}1&{}1&{}1\\ 1&{}1&{}1&{}1&{}1&{}1&{}1&{}1\\ 1&{}1&{}1&{}1&{}1&{}1&{}1&{}1\\ 1&{}1&{}1&{}1&{}1&{}1&{}1&{}1\\ 1&{}1&{}1&{}1&{}1&{}1&{}1&{}1\\ 1&{}1&{}1&{}1&{}1&{}1&{}1&{}1\\ 1&{}1&{}1&{}1&{}1&{}1&{}1&{}1\\ 1&{}1&{}1&{}1&{}1&{}1&{}1&{}1\\ \end{pmatrix} \end{aligned}$$

**Figure 11:**
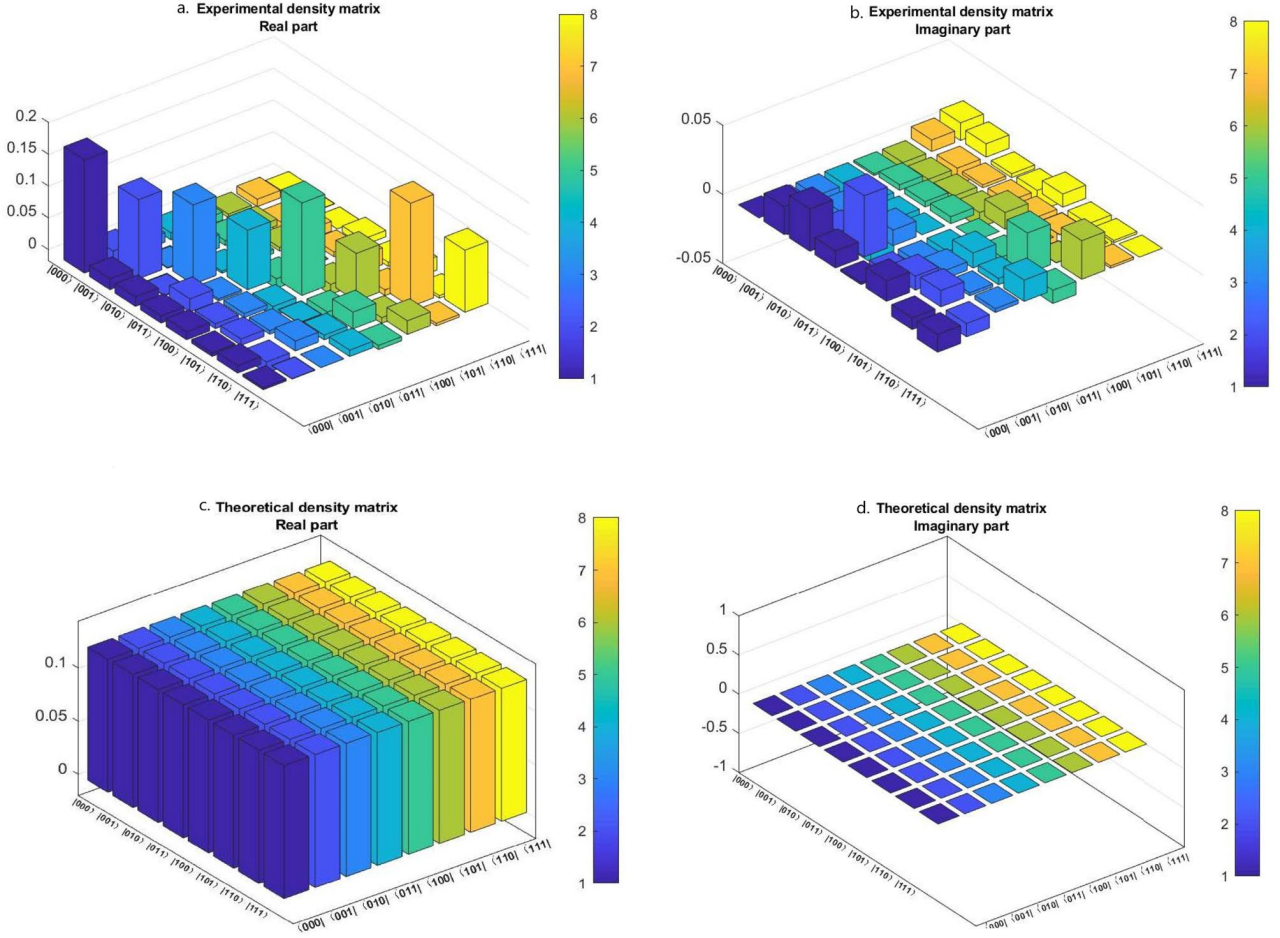
Real and imaginary parts of experimental and theoretical density matrices for teleportation of three-qubit state $| \chi \rangle _{abc}=\frac{1}{2\sqrt{2}}(| 000 \rangle +| 001 \rangle +| 010 \rangle +| 011 \rangle +| 100 \rangle +| 101 \rangle +| 110 \rangle +| 111 \rangle )_{abc}$. (**a**) Real part of the experimental density matrix, (**b**) imaginary part of the experimental density matrix, (**c**) real part of the theoretical density matrix, (**d**) imaginary part of the theoretical density matrix. These results are taken from the “IBM 16 Melbourne” device.

Besides, the experimental density matrix (18) for three-qubit is given by the following formula^67,93^

27 $$\begin{aligned} \rho ^E = \frac{1}{8}\sum _{j_1,j_2,j_3 =0}^{3}T_{j_1j_2j_3}(\sigma _{j_1}\otimes \sigma _{j_2}\otimes \sigma _{j_3}). \end{aligned}$$

where, $T_{j_1j_2j_3} =S_{j_1}\times S_{j_2}\times S_{j_3}$ and the Stokes parameters are $S_0 = P_{| 0I \rangle } + P_{| 1I \rangle }$, $S_1 = P_{| 0X \rangle } - P_{| 1X \rangle }$, $S_2 = P_{| 0Y \rangle } - P_{| 1Y \rangle }$, $S_3 = P_{| 0Z \rangle } - P_{| 1Z \rangle }$. Here, $P_{| 0j_1 \rangle }$represents the probability of the qubit to be found in $| 0 \rangle$ state when it is measured in $j_1$ basis. $P_{| 1j_1 \rangle }$ represents the probability of the qubit to be found in $| 1 \rangle$ state when it is measured in $j_1$ basis, and the same things with $j_2$ and $j_3$ bases. Hence, for the three-qubit state (25), the experimental density matrix is calculated as follows (see Fig. 11)

28 $$\begin{aligned} \rho ^E&= \begin{pmatrix} 0.1778 &{} 0.0172 &{} 0.0149 &{} 0.0112 &{} 0.0125 &{} 0.0056&{} 0.0116&{} 0.0031\\ 0.0172 &{} 0.1243&{} -0.0010&{} 0.0206&{} 0.0076 &{} 0.0100&{} -0.0174&{} -0.0006\\ 0.0149 &{} -0.0010 &{} 0.1245 &{} 0.0033&{} 0.0064 &{} 0.0056&{} 0.0155 &{} -0.0001\\ 0.0112 &{} 0.0206 &{} 0.0033 &{}0.0935&{} -0.0149 &{}0.0006&{} 0.0074 &{}0.0100\\ 0.0125 &{}0.0076 &{} 0.0064 &{}-0.0149 &{} 0.1452 &{}-0.0155 &{} 0.0261 &{}-0.0065\\ 0.0056 &{} 0.0100 &{} 0.0056 &{} 0.0006 &{} -0.0155 &{} 0.0747 &{} -0.0088 &{} 0.0214\\ 0.0116 &{} -0.0174 &{}0.0155 &{} 0.0074 &{} 0.0261 &{} -0.0088 &{} 0.1625 &{} -0.0040\\ 0.0031 &{}-0.0006 &{} -0.0001&{} 0.0100 &{}-0.0065&{} 0.0214 &{}-0.0040&{} 0.0975\\ \end{pmatrix} \\&\quad +i \begin{pmatrix} 0 &{} -0.0201 &{} -0.0306&{} -0.0122&{} -0.0014&{} -0.0148 &{} 0.0084&{} 0.0127\\ 0.0201 &{} 0 &{} -0.0060&{} -0.0441 &{} 0.0040 &{}-0.0101 &{} -0.0117 &{} 0.0096\\ 0.0306 &{}0.0060 &{} 0 &{} -0.0114&{} 0.0039 &{} -0.0060 &{} -0.0021 &{} -0.0027\\ 0.0122 &{}0.0441 &{}0.0114 &{} 0 &{}0.0050 &{} -0.0109&{} -0.0045 &{} -0.0154\\ 0.0014 &{} -0.0040 &{}-0.0039 &{}-0.0050 &{} 0 &{} 0.0121 &{}-0.0294 &{} 0.0095\\ 0.0148 &{} 0.0101 &{} 0.0060 &{}0.0109 &{} -0.0121 &{} 0 &{} 0.0002 &{} -0.0279\\ -0.0084 &{} 0.0117 &{} 0.0021&{} 0.0045 &{} 0.0294 &{} -0.0002 &{} 0 &{} -0.0016\\ -0.0127 &{}-0.0096&{} 0.0027 &{} 0.0154&{} -0.0095 &{} 0.0279&{} 0.0016 &{} 0\\ \end{pmatrix} \end{aligned}$$

Thereby, the fidelity between $\rho ^T$ and $\rho ^E$ is calculated to be 0.4006.

## Security analysis against Charlie

We perform a security analysis against Charlie to check his honesty. In the security analysis protocol, Bob has to contact Alice through a classical channel, as well as he has to measure his state in the basis decided by Alice. For realizing security analysis of our protocols, we have taken Bell state and GHZ state as a two-qubit state and three-qubit state respectively for teleportation.

### For teleportation of two-qubit state

Now, Alice wants to share a Bell state as $| \psi ^- \rangle =\frac{1}{\sqrt{2}}(| 01 \rangle -| 10 \rangle )$, and after performing all the unitary transformations in a systematic way as discussed before, Eq. (7) can be rewritten as

29 $$\begin{aligned} | \psi ''' \rangle _{ab12345}&= \frac{1}{4}(| 0000 \rangle +| 0001 \rangle +| 0010 \rangle +| 0011 \rangle +| 0100 \rangle +| 0101 \rangle +| 0110 \rangle \\&\quad +| 0111 \rangle +| 1000 \rangle +| 1001 \rangle +| 1010 \rangle +| 1011 \rangle +| 1100 \rangle +| 1101 \rangle \\&\quad +| 1110 \rangle +| 1111 \rangle )_{ab13}\otimes \frac{1}{\sqrt{2}} \left( \frac{1}{\sqrt{2}}(| 01 \rangle -| 10 \rangle )_{25}\otimes | + \rangle _{4} +\frac{1}{\sqrt{2}}(-| 01 \rangle +| 10 \rangle )_{25}\otimes | - \rangle _{4}\right) . \end{aligned}$$

In this case, Bob has to measure his state in Bell basis. Now, Alice measures her states and let us say she gets $| 0000 \rangle$ then the whole state is get collapsed to $\frac{1}{\sqrt{2}}(\frac{1}{\sqrt{2}}(| 01 \rangle -| 10 \rangle )_{25}\otimes | + \rangle _{4} +\frac{1}{\sqrt{2}}(-| 01 \rangle +| 10 \rangle )_{25}\otimes | - \rangle _{4})$. Next, Charlie measures his state and let us say that the measurement outcome is $| + \rangle _{4}$. Now, in this case, Charlie does not need to send classical information to Bob as discussed earlier. Suppose, Charlie is a dishonest person and he sends the one bit of classical information to Bob within a time period. As usual, after receiving the information, Bob gets that Charlie’s qubit is in $| - \rangle _{4}$ state and he performs the phase-change unitary transformation on his qubits. After performing the unitary transformation, Bob measures his state in Bell basis and he gets $| Bob-state \rangle =-\frac{1}{\sqrt{2}}(| 01 \rangle -| 10 \rangle )$. Then he contacts Alice and they both exchange their information about the state and then they both know that Charlie cheated.

### For teleportation of three-qubit state

In this case, suppose Alice wants to share a GHZ state given as $| GHZ \rangle =\frac{1}{\sqrt{2}}(| 010 \rangle -| 101 \rangle )$, and after performing all the unitary transformations as discussed above in a systematic way, Eq. (13) becomes

30 $$\begin{aligned} | \chi '' \rangle _{abc1234567}&= \frac{1}{8}\sum _{l=0}^{l=63}| \chi _l \rangle _{abc135}\otimes \frac{1}{\sqrt{2}} \\&\quad \left( \frac{1}{\sqrt{2}}(| 011 \rangle - | 110 \rangle )_{246}\otimes | + \rangle _{7} + \frac{1}{\sqrt{2}}(-| 011 \rangle -| 110 \rangle )_{246}\otimes | - \rangle _{7}\right) . \end{aligned}$$

In this case, Bob has to measure his state in GHZ basis^94,95^. Now, Alice measures her states and let us say she gets $| \chi _{0} \rangle$ then the whole state is get collapsed to $\frac{1}{\sqrt{2}}(\frac{1}{\sqrt{2}}(| 011 \rangle -| 110 \rangle )_{246}\otimes | + \rangle _{7} + \frac{1}{\sqrt{2}}(-| 011 \rangle -| 110 \rangle )_{246}\otimes | - \rangle _{7})$. Next, Charlie measures his state and let us say that the measurement outcome is $| + \rangle _{7}$. Now Charlie does not need to send classical information to Bob as discussed earlier. Suppose, Charlie is a dishonest person and he sends the one bit of classical information to Bob within a time period. As usual, after receiving the information, Bob gets that Charlie’s qubit is in $| - \rangle _{7}$ state. Then, Bob first performs the phase-change unitary transformation on his qubit 6 and then he applies CNOT gate on his qubits (4, 6) and (2, 4). After performing the unitary transformations, Bob measures his state in GHZ basis and he gets $| Bob-state \rangle =\frac{1}{\sqrt{2}}(-| 010 \rangle -| 101 \rangle )$. Then Bob communicates with Alice and they both exchange their information about the state and thereby they know that Charlie cheated.

## Conclusions

In this work, we theoretically and experimentally demonstrate the teleportation of two-qubit and three-qubit states through five-qubit and seven-qubit cluster states respectively. As shown in the circuit diagrams, we successfully run those circuits on “IBM qasm simulator” as well as on “IBM 16 Melbourne” and report their probability distribution results. And we show that the teleportation of qubits through five-qubit and seven-qubit cluster states is possible. We also calculate the fidelity for both the cases and obtain a genuine fidelity over 40%. Remarkably, we also examine the security analysis against Charlie, and these schemes which we consider here are secure against Charlie’s attacks. We show that the average classical communication cost of our protocols is 0.5 bit. As compared to other protocols, our protocols reduce substantially the classical communication cost. Hence, our protocols are economical and achievable.

*Note* The circuits shown in this work are drawn on the IBM circuit drawer and they are equivalent to all the operations discussed in the text. Confusion might create between the “circuits operations” and “Bob’s operations”. Here, when Charlie measurement outcome is $| \pm \rangle$, then Bob has to perform the unitary operations as shown in Tables 2 and 3. However, as seen from quantum circuits in Figs. 4 and 6, it is shown that Hadamard gate is applied on Charlie’s qubit and CZ operations are applied between Charlie’s qubit and Bob’s qubits. These operations are just for convenience to get the appropriate results.

## Data Availability

The data that support the findings of this study are available from the authors (akabhijeet200396@gmail.com, bikash@bikashsquantum.com) upon reasonable request.
